# Specific protein-RNA interactions are mostly preserved in biomolecular condensates

**DOI:** 10.1126/sciadv.adm7435

**Published:** 2024-03-06

**Authors:** Tebbe de Vries, Mihajlo Novakovic, Yinan Ni, Izabela Smok, Clara Inghelram, Maria Bikaki, Chris P. Sarnowski, Yaning Han, Leonidas Emmanouilidis, Giacomo Padroni, Alexander Leitner, Frédéric H.-T. Allain

**Affiliations:** ^1^ Department of Biology, Institute of Biochemistry, ETH Zurich, Zurich, Switzerland.; ^2^ Department of Biology, Institute of Molecular Systems Biology, ETH Zurich, Zurich, Switzerland.

## Abstract

Many biomolecular condensates are enriched in and depend on RNAs and RNA binding proteins (RBPs). So far, only a few studies have addressed the characterization of the intermolecular interactions responsible for liquid-liquid phase separation (LLPS) and the impact of condensation on RBPs and RNAs. Here, we present an approach to study protein-RNA interactions inside biomolecular condensates by applying cross-linking of isotope labeled RNA and tandem mass spectrometry to phase-separating systems (LLPS-CLIR-MS). LLPS-CLIR-MS enables the characterization of intermolecular interactions present within biomolecular condensates at residue-specific resolution and allows a comparison with the same complexes in the dispersed phase. We observe that sequence-specific RBP-RNA interactions present in the dispersed phase are generally maintained inside condensates. In addition, LLPS-CLIR-MS identifies structural alterations at the protein-RNA interfaces, including additional unspecific contacts in the condensed phase. Our approach offers a procedure to derive structural information of protein-RNA complexes within biomolecular condensates that could be critical for integrative structural modeling of ribonucleoproteins (RNPs) in this form.

## Teaser

UV cross-linking and mass spectrometry provide structural insights into protein-RNA interactions within biomolecular condensates.

## INTRODUCTION

Membraneless cellular bodies such as the nucleoli or stress granules are generated via liquid-liquid phase separation (LLPS) (*1*, *2*). LLPS is a molecular mechanism that leads to a density transition in a super-saturated milieu that drives the solution to separate into two distinct phases, the condensed phase and the dispersed phase. The formation of membraneless organelles via LLPS promotes the compartmentalization of cellular matter and thus enables spatiotemporal regulation of biological reactions (*3*, *4*). An ever-increasing number of studies have demonstrated the essential role of LLPS in diverse biological activities, including gene expression processes such as splicing, 3′-end processing, miRNA processing, and translation. Multiple neurodegenerative diseases, such as amyotrophic lateral sclerosis (ALS) and frontotemporal lobar degeneration, result from the accumulation of irreversible aggregates that may be formed via LLPS (*5*, *6*). The irreversible phase transition is enhanced by disease-associated mutations in RNA binding proteins (RBPs), such as in the case of Fused in Sarcoma (FUS) that has been shown to aggregate into a solid state (liquid-to-solid transition) (*7*, *8*). Proteins containing repetitive amino acid sequences, so-called low complexity domains (LCDs), often show a high degree of conformational flexibility [referred to as intrinsically disordered regions (IDR)] and promote LLPS via a combination of promiscuous intermolecular interactions, including unspecific hydrophobic, stacking, and hydrogen bonding interactions (*9*–*11*). Structural studies by solution state nuclear magnetic resonance (NMR) spectroscopy of the LCDs of FUS, DDX4, hnRNPA2, and CAPRIN1 demonstrated that all retain disorder within the condensed phase (*12*–*15*); however, the FUS LCD shows a slight compaction in the condensed phase (*16*).

There is accumulating evidence that many biomolecular condensates depend on RNAs and RBPs, both of which undergo phase separation individually or in combination (*17*–*19*). RBPs are often composed of structured RNA binding domains (RBDs) interspersed by IDRs. While the role of IDRs of some RBPs in condensates has been extensively studied, the function of RNA and the interaction with the RBPs, in particular their folded RBDs, inside condensates has not been well explored. Thus far, the molecular understanding of the physicochemical properties of RNA in condensates is limited. Some studies have suggested a suppressive effect of RNA on RBP phase separation (*20*), while generally the anionic properties of RNA are thought to promote nonspecific protein-RNA interactions that contribute to condensation. In protein-RNA mixtures, the concentration of RNAs is important for LLPS as low concentrations of RNA support the formation of condensates and above a certain threshold, increasing concentrations lead to the dissolution of the condensates, as a result of a charge inversion in the phase-separated system (*20*, *21*). This effect has been described as reentrant phase behavior and has recently been implicated in transcriptional regulation (*22*).

General driving forces of LLPS are homotypic (e.g., protein-protein) or heterotypic (e.g., protein-RNA) multivalent interactions between the participating biomolecules (*23*). Multivalent interactions between biopolymers reduce the average distance between them, thus producing a much denser phase than the aqueous one. Recent studies suggest that besides charge interactions, specific interactions between RNA targets and RBPs contribute to multivalency that promotes LLPS (*24*–*26*).

However, it remains largely unknown how condensation influences interactions of RNAs and proteins. Recently, in an individual nucleotide resolution ultraviolet (UV) cross-linking and immunoprecipitation (iCLIP) study using various TDP-43 mutants with diverse condensation properties, it was found that the binding to specific RNA regions varies across the transcriptome depending on the condensation properties of each mutant (*27*). Also, using RNP-MaP (ribonucleoprotein networks analyzed by mutational profiling) (*28*), which uses a lysine-specific protein-RNA cross-linking agent, a different mapping of severe acute respiratory syndrome coronavirus 2 (SARS-CoV-2) nucleocapsid protein on viral RNA was identified under conditions of LLPS as compared to the dispersed state (*29*).

Although phase separation can be investigated in vitro through the formation of liquid droplets (*30*), to our knowledge, the structure determination of protein-RNA complexes in the condensed phase, as well as the detailed characterization of the intermolecular interactions responsible for phase separation of such complexes, has not been achieved, largely due to the lack of appropriate methods (*10*, *31*). Here, we present a method to elucidate the protein-RNA interactions present inside such biomolecular condensates, which is based on protein-RNA cross-linking with UV light followed by characterization of the cross-linking products of phase-separated systems by mass spectrometry. We applied this method to several RBP-RNA complexes under conditions where the condensed and dispersed phases coexist, distinguishing it from previous application to dispersed systems (*32*–*35*). With this method, we demonstrate that sequence-specific interactions of RBDs with RNA are preserved inside condensates. In addition, we identify subtle structural alterations at protein-RNA interfaces between the two phases, including more unspecific protein-RNA interactions in the condensed phase. Our approach offers a procedure to derive structural information of protein-RNA complexes within biomolecular condensates and could be used to generate integrative structural models of ribonucleoproteins (RNPs) in this form.

## RESULTS

### The LLPS-CLIR-MS method

**Fig. 1.**
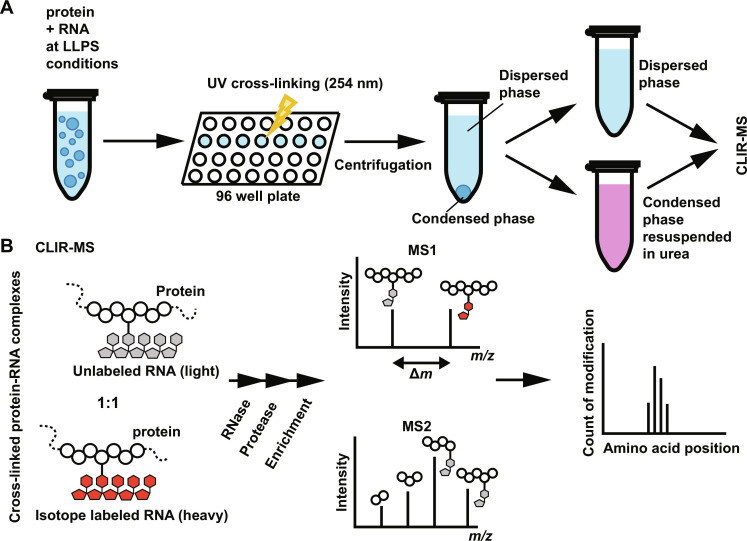
The LLPS-CLIR-MS approach. (**A**) A droplet-forming solution of RNA and RBP is irradiated with UV light (254 nm) and centrifuged to separate the condensed from the dispersed phase. The cross-linked protein-RNA complexes of the condensed phase are resuspended in buffer containing urea. (**B**) For CLIR-MS, a mixture of heavy and light RNA is used to facilitate the detection of protein-RNA cross-links by MS. Proteins and RNAs are enzymatically digested and protein-RNA cross-links are enriched (see Materials and Methods). Peptide-oligonucleotide adducts are separated by LC and ionized, and their mass/charge ratio (*m/z*) is determined, with protein-RNA cross-links appearing as doublets with a specific mass shift (Δ*m*) that depends on the type and composition of covalently linked oligonucleotide(s) and the isotope labeling scheme. Abundant peptide-RNA adducts are fragmented to determine the position and composition of the covalently linked RNA adduct (but not the sequence). Protein-RNA cross-links can be mapped back on the sequence of the protein.

Interactions between amino acid side chains in proteins and RNA nucleobases can be covalently stabilized upon irradiation with UV light. Our previously introduced CLIR-MS (cross-linking of isotope labeled RNA coupled to mass spectrometry) method (*32*) combines stable isotope labeling of RNA with cross-linking of protein-RNA interactions at 254 nm and subsequent liquid chromatography–tandem mass spectrometry (LC-MS/MS) analysis. Through the identification of protein-RNA cross-links, the method enables the characterization of the RNA binding interface at single amino acid and single nucleotide resolution. In this study, we developed a specific workflow for biomolecular condensates, which we termed LLPS-CLIR-MS. The new methodology was applied to phase-separated protein-RNA complexes by UV cross-linking simultaneously both in the condensed (droplets) and dispersed phases. Both phases are then separated by centrifugation (Fig. 1A) and subjected to independent analysis by the CLIR-MS pipeline that is presented in Fig. 1B.

### Presence of sequence-specific contacts within PTBP1-RNA condensates

**Fig. 2.**
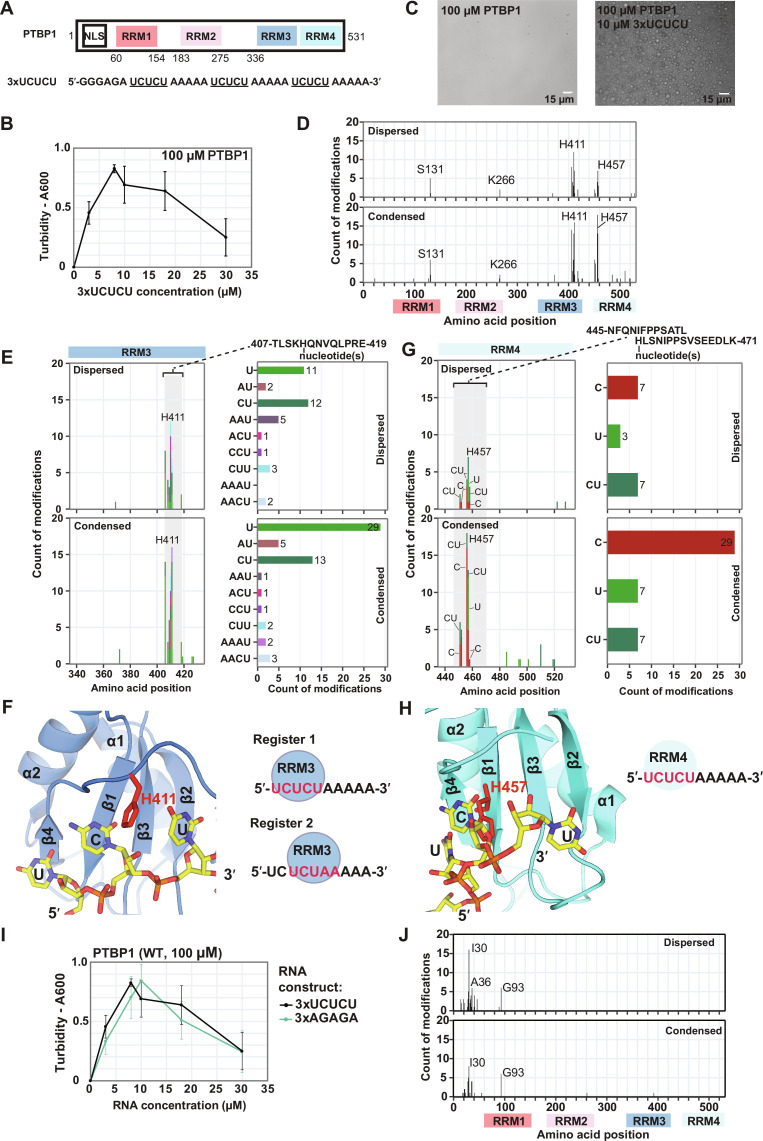
PTBP1 binds sequence-specifically to RNA inside droplets. (**A**) Domain scheme of PTBP1 and sequence of 3xUCUCU RNA. NLS, nuclear localization signal; RRM, RNA recognition motif. (**B**) Turbidity measurement (absorbance at 600 nm) of PTBP1 and 3xUCUCU. 3xUCUCU induces condensation of PTBP1 at substoichiometric concentrations and increasing concentrations dissolve droplets. (**C**) Light microscopy of free PTBP1 (left) and PTBP1 forming droplets in the presence of 3xUCUCU (right). (**D**) Total number of protein-RNA cross-link identifications plotted on the sequence of PTBP1. (**E**) H411 cross-links and attached nucleotide modifications. (**F**) Solution structure of RRM3 bound to 5′-UCU-3′. RRM3 can adopt two different registers in complex with 3xUCUCU. (**G**) H457 cross-links and attached nucleotide modifications. (**H**) RRM4 adopts a single register in complex with 3xUCUCU. (**I**) Turbidity measurements of PTBP1 and 3xUCUCU and 3xAGAGA, respectively. (**J**) Cross-links detected in LLPS-CLIR-MS experiment of PTBP1 with 3xAGAGA RNA imply that predominantly unspecific interactions can also induce phase separation of PTBP1. The error bars in (B) and (I) represent the SDs of the three technical replicates.

We first established the LLPS-CLIR-MS approach on condensates containing the RBP polypyrimidine tract binding protein 1 (PTBP1), which features four folded RNA-recognition motifs (RRM), of which RRM1, RRM2, and RRM3 are each separated by flexible linkers, while RRM3 and RRM4 adopt a rigid conformation by interacting with each other (Fig. 2A) (*36*). PTBP1 is soluble at high, up to millimolar concentrations, and LLPS is not observed for the free protein in the absence of RNA. However, PTBP1 readily phase-separates upon addition of substoichiometric amounts of an RNA containing three repeats of a 5′-UCUCU-3′ motif separated by adenosines (referred to as 3xUCUCU) as monitored by an increase in turbidity and by observing droplet formation using light microscopy (Fig. 2, B and C), similarly as shown previously by others (*23*). PTBP1 phase separation reaches a maximum at a 1:10 RNA-to-protein ratio (100 μM protein concentration, 10 μM RNA concentration) as evidenced by turbidity measurements. Higher concentrations of RNA lead to dissociation of these droplets. This behavior is typical for systems showing reentrant phase transitions (*21*). To determine whether the interaction of PTBP1 with RNA is exclusively unspecific and based on charge interactions, as proposed for reentrant phase transitions, or if specific protein-RNA contacts are maintained inside condensates, we applied LLPS-CLIR-MS to the PTBP1/3xUCUCU complex. The structural details of the interaction of each domain of PTBP1 in complex with 5′-UCUCU-3′ have been well characterized in solution (*37*). Phase separation was induced by addition of 3xUCUCU RNA (50% ^13^C/^15^N-labeled, 50% unlabeled) to PTBP1 at a defined ratio to obtain a mixture of dispersed and condensed complexes of equal amounts (for details see Materials and Methods). These samples were cross-linked by irradiation with UV light that results in the formation of a covalent bond between RNA nucleotides and amino acid residues within close proximity, found or known to predominantly involve π-stacking (*38*). The two phases were subsequently separated by centrifugation. The supernatant (dispersed phase) was carefully removed, and the condensed phase, which was present as a pellet, was resuspended in urea-containing buffer. The presence of cross-linked protein-RNA complexes in both phases was confirmed by SDS gel analysis of the respective samples (fig. S1A). Subsequently, the established CLIR-MS protocol was applied involving enzymatic digestion of the protein and RNA with protease and nonspecific nucleases to generate specific peptide sequences cross-linked to short oligonucleotide fragments, followed by additional purification and enrichment steps and LC-MS/MS analysis (*34*). The use of differentially isotope-labeled RNA improves the detection of cross-linked peptides by MS as they generate characteristic isotope patterns (“doublets”) in the precursor ion mass spectrum (*32*, *39*). The peptide component of peptide-RNA conjugates is then fragmented, facilitating determination of both the position and composition of the covalently linked RNA adduct (but not the sequence).

By applying the LLPS CLIR-MS approach on PTBP1/3xUCUCU, we observed strong similarities in cross-linking patterns between the dispersed and condensed phase sample. In the system studied here, cross-links were detected for all four RRMs of PTBP1, and the most abundant cross-linking sites (by spectral count) were protein residues surrounding H411 (RRM3) and H457 (RRM4) (Fig. 2D, all MS identifications are compiled in table S1), consistent with previous reports (*32*, *38*). These residues are important for specific RNA recognition and are integral parts of the protein-RNA interface of the PTBP1 RRM domains (*37*). The most frequently cross-linked nucleotides were uridines and a uridine-cytosine di-nucleotide, consistent with a sequence-specific binding to the 5′-UCUCU-3′ motif. These cross-linking sites were present in both the dispersed and condensed phases. The identification of identical cross-links in both phases suggests that the molecular contacts observed in the dispersed phase are also present in the condensed phase, thus indicating the presence of sequence-specific protein-RNA contacts inside condensates involving the folded RRMs.

The most abundantly detected cross-link in the diluted and condensed phase was formed between residues at or close to histidine H411 and uridine (Fig. 2E), indicating a stacking interaction, as expected from the structure of RRM3 bound to RNA (Fig. 2F). However, many cross-links of H411 also involve nucleotide fragments containing adenosines. This implies that the interactions in the protein-RNA complex can adopt additional registers. RRM3, which has an RNA binding specificity for 5′-YCUNN-3′ (Y: pyrimidine, N: any nucleotide) binds the 5′-UCUCU-3′ sequence (register 1) but can also bind the 5′-UCUAA-3′ sequence (register 2) (Fig. 2F). This illustrates that the approach can also probe binding to multiple binding registers. In contrast, RRM4, which has a RNA binding specificity of 5′-YCN-3′, binds exclusively to the 5′-UCUCU-3′ sequence, as suggested by the nucleotides cross-linked to H457 (Fig. 2, G and H). Therefore, specific binding sites and potentially a tolerance for additional RNA binding registers, as suggested for RRM3, are seen in both phases.

To exclude that similar cross-linking patterns were detected due to exchange of molecules between the dispersed and condensed phase during cross-linking, we analyzed a sample of PTBP1/3xUCUCU in which the two phases were separated before UV cross-linking (fig. S2). Also, in this case, the same cross-linking patterns were detectable in the dispersed and condensed phases.

To validate the importance of specific binding sites for LLPS, we analyzed the phase separation properties of PTBP1 with mutations in the RRM domains, which prevent (sequence-specific) RNA binding (RRM3 mut: F371A, K374A; RRM4 mut: H457A, R523A, K528A). Similar to the wild-type (WT) protein, both RRM3 and RRM4 mutants display RNA-dependent complex coacervation and reentrant-phase separation behavior (fig. S3A). It is noteworthy that in the case of the WT protein, RNA concentrations of 30 to 50 μM cause droplets to gradually disperse. However, the mutant proteins RRM3 and RRM4 exhibit notable phase separation at these concentrations. This behavior might arise because the mutations weaken affinity and sequence-specific RNA binding (*40*), preventing rapid RNA saturation by PTBP1 and ultimately leading to sustained high levels of phase separation. This suggests that by interfering with sequence-specific interactions, changes are introduced to the phase separation characteristics of PTBP1, primarily affecting the disassembly of condensates rather than their initial formation. Similarly, we then studied a nonspecific RNA containing three repeats of a 5′-AGAGA-3′ motif instead of the specific UCUCU motifs (referred to as 3xAGAGA). Quite unexpectedly, we observed a very similar phase separation behavior as with 3xUCUCU when incubated with PTBP1 (Fig. 2I and fig. S3, B and C). However, in contrast to 3xUCUCU RNA, cross-linking to the RRMs was not observed anymore (Fig. 2J). This implies that the level of droplet formation by PTBP1 does not necessarily correlate with the RNA binding specificity. These data also revealed that the N-terminal region of PTBP1 can bind noncognate RNA unlike the RRMs. Therefore, these data illustrate that both sequence-specific and unspecific contacts can contribute to RNA-induced LLPS.

PTBP1 also shows typical reentrant phase separation behavior with structured RNAs, such as the SL4 of U1 snRNA (U1-SL4), which is bound by PTBP1 during splicing repression (fig. S4, A and B) (*41*, *42*). Using LLPS-CLIR-MS, we found consistent cross-linking patterns for the PTBP1/U1-SL4 complex in both dispersed and condensed phase (figs. S1B and S4, C to G), thus supporting the presence of specific contacts between RNA and protein in the condensed phase also when bound to structured RNA molecules.

Overall, instead of being driven solely by charge interactions, the mechanism of LLPS for the PTBP1/3xUCUCU system depends also on sequence-specific, multisite protein-RNA interactions, in which the RRMs from different protein copies interact multivalently with various binding sites in the RNA chain, thus forming a dynamically interconnected biomolecular network. The tolerance of additional registers of RRM3 may contribute to this multivalency.

### Involvement in RNA binding by both folded and disordered domains in FUS condensates

**Fig. 3.**
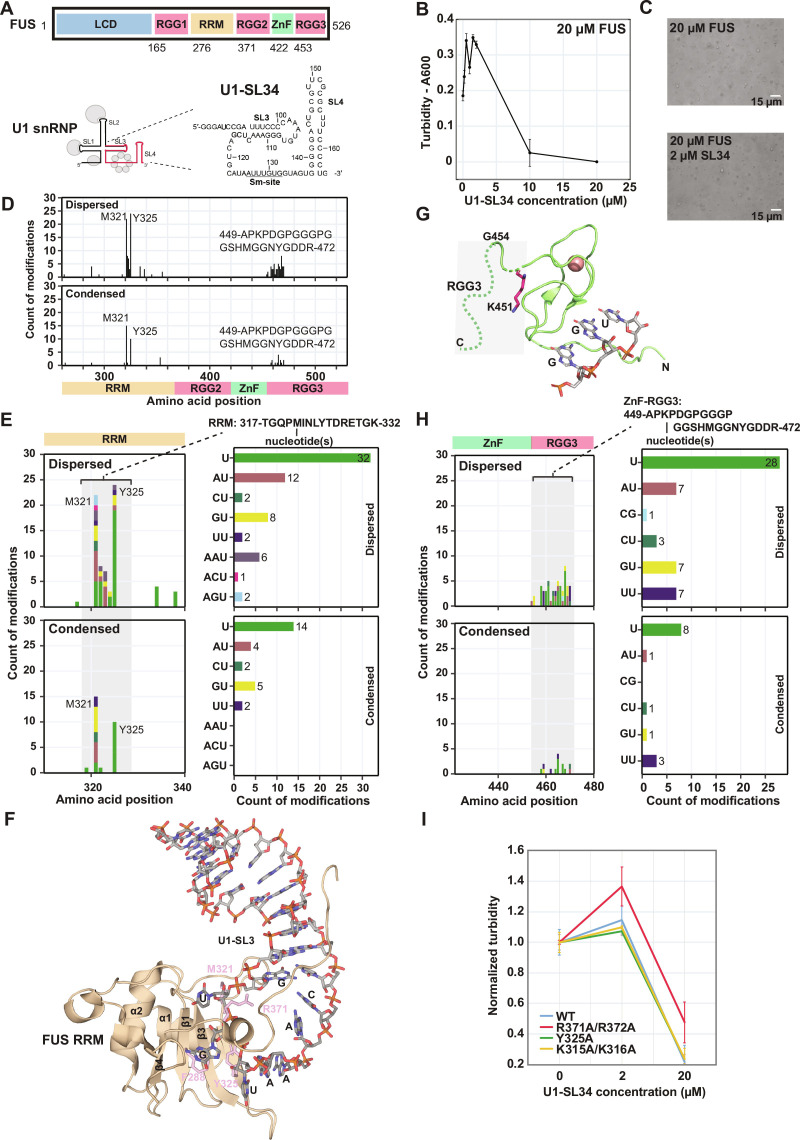
FUS RRM and IDR bind sequence-specifically to U1-SL34. (**A**) Domain scheme of FUS and sequence and secondary structure of U1-SL34. (**B**) Turbidity measurements of (full-length) FUS and U1-SL34. (**C**) Light microscopy of FUS alone and with U1-SL34. (**D**) Overview of the protein-RNA cross-links detected for FUS and U1-SL34 by LLPS-CLIR-MS. (**E**) Main cross-linking site of FUS RRM (M321 and Y325) and the respective cross-linked nucleotides. (**F**) Cross-links detected for FUS RRM fit well with the solution structure of the protein in complex with U1-SL3. (**G**) The region C-terminal of the zinc finger domain illustrated on the solution structure of this domain in complex with 5′-GGU-3′. RGG3 domain is highlighted in the structure. (**H**) The region C-terminal to the zinc finger domain cross-links to a diverse set of nucleotides. (**I**) Turbidity measurements of FUS RNA binding mutants and U1-SL34. Mutation of the RNA binding interface alters LLPS of FUS. The error bars in (B) and (I) represent the SDs of the three technical replicates.

To validate that specific protein-RNA interactions are involved in the multivalent network inside condensates, we also applied the LLPS-CLIR-MS approach on a sample containing full-length FUS. FUS consists of an LCD, three RGG boxes, one RRM, and a zinc finger domain (ZnF) (Fig. 3A). In contrast to the PTBP1 discussed above, FUS by itself undergoes LLPS at room temperature, as the N-terminal LCD and RGG-rich segments favor demixing from aqueous solutions (*15*). CLIP studies showed that FUS can bind stem-loop 3 (SL3), stem-loop 4 (SL4), and the Sm site of U1 snRNA (*43*, *44*). We have previously characterized the structure of the RRM of FUS in complex with SL3 and binding of FUS-ZnF to a 5′-GGU-3′ sequence that is also present in the Sm site (*43*, *45*). Binding of the ZnF to the Sm site was proposed as a mechanism for aggregation formation in ALS by trapping small nuclear ribonucleoproteins biogenesis intermediates in human and murine motor neurons (*43*). Despite formation of droplets in the absence of RNA through homotypic interactions, FUS shows a slight increase of phase separation in the presence of substoichiometric amounts of structured RNA (U1-SL34) (up to 0.1 equivalents of U1-SL34 in 20 μM FUS) (Fig. 3B,C), most likely by increasing the effective valency of FUS within the condensate (*46*). By applying LLPS-CLIR-MS on the sample containing 0.075 equivalents of U1-SL34, we identified cross-links in the RRM of FUS and in the region C-terminal to the ZnF close to an RGG motif (RGG3) (Fig. 3D). Cross-links at the same amino acid positions were identified both in the dispersed and condensed phases. The identity of the nucleotides cross-linked to residues of the RRM corresponds well with a mapping on SL3 (Fig. 3, E and F). These cross-links are preserved also in the FUS sample prepared at RNA/protein ratio of 0.1:1 as illustrated in fig. S5. These data show that similarly to PTBP1, specific protein-RNA contacts are formed in the condensed form. Consistent with the PTBP1 results shown above, mutating residues affecting the RNA binding affinity of the RRM (Y325, K315/K316, and R371/R372 to alanine) did not influence phase separation drastically as shown by both increased or comparable turbidity values compared to the WT protein (Fig. 3I). The C-terminal region downstream of the ZnF is an IDR and revealed unexpected cross-links (Fig. 3H). This indicates that the sequence C-terminal to the ZnF also contributes to RNA binding and that this part of the protein may rigidify upon SL34 binding, to allow the cross-linking reaction to occur (*38*). The proline-glycine–rich sequence is followed by an RGG box, which might bind to another site of the RNA, such as SL4, potentially through an interaction similar to the one recently observed for the RGG motif of SF3A1 in complex with SL4 (*33*). This could bring the glycine-proline–rich sequence close to RNA, which results in the detected cross-links. The interaction of this IDR was independent of condensation of the complex. This shows that the approach can also capture protein-RNA interactions that occur in IDRs. Overall, the data show that specific protein-RNA contacts formed in the dispersed state are also present in condensates to enhance FUS LLPS.

### Unspecific protein-RNA interactions of SARS-CoV-2 nucleocapsid protein

**Fig. 4.**
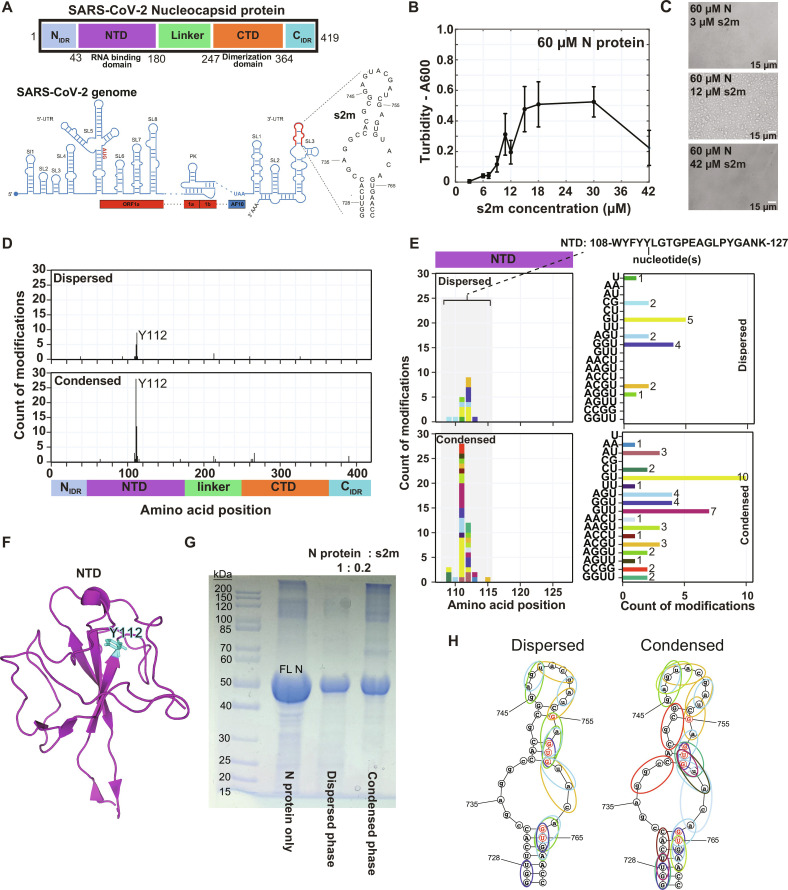
Nucleocapsid interaction with s2m is less specific within condensates. (**A**) Domain scheme of SARS-CoV-2 nucleocapsid protein and sequence and secondary structure of s2m. (**B**) Turbidity measurements of N protein and s2m RNA at room temperature. The error bars represent the SD of the three technical replicates. (**C**) Light microscopy of N protein and s2m in different ratios. (**D** and **E**) Overview of the protein-RNA cross-links detected for nucleocapsid and s2m by LLPS-CLIR-MS. (**F**) The most abundant cross-linked amino acid, Y112, shown on the structure of the NTD (PDB ID 7CDZ). (**G**) Denaturing gel showing that dispersed and condensed phase contain similar amounts of nucleocapsid protein upon cross-linking for a given protein:RNA stoichiometry. As a reference, N protein without RNA upon cross-linking is shown. (**H**) Mapping of the RNA binding site of nucleocapsid on s2m. Cross-linked nucleotide modifications (longer than 2 nucleotides) were illustrated using ellipsoids using color code from (E). The combination of cross-linked nucleotides suggests an altered interaction of the NTD with s2m in the condensed state. This is corroborated by additional CLIR-MS experiments using samples mimicking completely dispersed and condensed phases (fig. S7). Chemical shift perturbations of imino proton peaks of s2m upon titration of nucleocapsid-NTD showed that the NTD preferably binds the region G755 to U765 of s2m (nucleotides highlighted in red) (*35*).

Last, the SARS-CoV-2 nucleocapsid protein (N protein) was assessed in complex with s2m RNA of the viral 3′ untranslated region (3′-UTR; Fig. 4A). N protein comprises an N-terminal disordered region (N_IDR_), a folded N-terminal domain (NTD) that participates in RNA binding, a linker (linker_IDR_), a C-terminal dimerization domain (CTD), and a C-terminal disordered tail region (C_IDR_). Nucleocapsid protein can undergo LLPS by itself at higher temperatures (*29*); however, addition of a structured RNA, such as the s2m RNA, from the 3′-UTR of SARS-CoV-2 genome, greatly promotes phase separation at room temperature (Fig. 4, B and C). Robust phase separation is achieved upon addition of ~0.2 to 0.4 equivalents of s2m, and droplets dissolve upon higher concentrations, thereby showing a similar reentrant phase transition as PTBP1.

In contrast to the previously introduced protein-RNA complexes, differential isotope labeling for LLPS-CLIR-MS was performed after protein and RNA digestion (postdigestion labeling) through enzymatic conjugation of ^18^O_4_–γ–adenosine triphosphate (ATP) or ATP to the 5′-hydroxyl groups of cross-linked oligonucleotides (*47*). The most abundant cross-linking site (by spectral count) was identified in the NTD around the aromatic residues Y109 and Y111, Y112 and L113, and G114 and T115 on the β3 strand of the NTD (Fig. 4, D to F). These residues have been identified as important for RNA binding by NMR spectroscopy and a Y109A mutant showed altered RNA binding (*29*, *48*). In contrast to the published model of individual NTD domain binding to single and double-stranded RNA (*48*), the detected cross-links suggest sequence specificity to GUG motifs, which is in agreement with independent NMR observations (*35*). Despite very similar quantity of the nucleocapsid protein in the two phases (Fig. 4G), detailed analysis of the detected nucleotide modifications on s2m RNA reveals substantial differences between the two phases in the s2m sites that are in contact with the nucleocapsid (Fig. 4H). In the dispersed phase, the most abundant cross-linked oligonucleotides are GU and GGU, suggesting binding to G757-U758-G759 and G764-U765-G766, which is consistent with NMR chemical shift perturbations of s2m upon titration with the NTD subdomain in non-LLPS conditions (*35*). However, in the condensed phase, the mapping of nucleotide modifications onto the s2m structure implies many additional interactions between s2m and the nucleocapsid protein (i.e., GUU or AAGU for example), in addition to the aforementioned specific ones (Fig. 4E). This difference suggests that in the condensed phase, the nucleocapsid would bind more unspecifically and may potentially break the stem-loop structure since we detect evidence of protein-RNA cross-links on both sides of the RNA stem and internal loop (Fig. 4H).

Using NMR spectroscopy, we found additional evidence for unspecific nucleocapsid-RNA interactions within liquid droplets. To examine the specificity of interactions, we performed binding experiments with N protein and two s2m RNA sequences that were extended by a polyA or a polyC tail (referred to as s2m-polyA and s2m-polyC, respectively). Both RNAs showed increased phase separation behavior compared to s2m WT indicating an involvement of the RNA 3′ tails in LLPS (fig. S6A). Next, we found that the NMR peaks corresponding to the polyA tail (10 nucleotides) were unobservable at a 0.2 to 1 RNA:protein ratio at a condition that promotes LLPS (fig. S6B). However, at a 1:1 ratio, when the droplets dissolve, adenine H2 and H8 proton peaks of the polyA tail were the only RNA peaks observable in the NMR spectrum (fig. S6C). This shows that the polyA tail interacts with the nucleocapsid in the condensed state but not in the dispersed state. With s2m-polyC (30 nucleotides), we prepared a phase separated sample of a complex with the nucleocapsid stabilized in 0.5% agarose (*10*) at 0.35:1 RNA to protein. Here, pyrimidine H5/H6 proton correlations of the polyC tail were detected, while no correlations corresponding to other pyrimidines from the RNA were observed (fig. S6D). To distinguish whether the H5/H6 proton correlations of the polyC tail originate from the RNA inside the droplets (condensed phase) or in the dispersed phase, we performed an NMR DOSY (Diffusion Ordered SpectroscopY) experiment (fig. S6E) (*10*). DOSY (*49*) editing technique can be used to spectrally select molecules based on their different diffusion constants and therefore can be applied to differentiate protein-RNA complexes inside and outside the droplets. Upon application of a strong gradient when only slow-diffusing species from the condensed phase should be observed, no H5/H6 proton peaks could be detected. This implies that the H5/H6 proton signals of the polyC originate from molecules present in the dispersed phase, where specific protein-RNA interactions dominate and the polyC tail is not bound. In the condensed phase, the H5/H6 proton correlations are unobservable most likely because of unspecific protein-RNA interactions with the polyC tail under these conditions resulting in the signal of the bound polyC tail being broadened beyond detection.

In conclusion, these NMR experiments support independently the LLPS-CLIR-MS results, namely that the SARS-CoV-2 nucleocapsid binds RNA less specifically in the condensed phase than in the dispersed phase.

## DISCUSSION

Together, the LLPS-CLIR-MS method presented here enables the characterization of intermolecular interactions between RNA and protein that are present within biomolecular condensates at site-specific resolution. Our data imply that RNAs in LLPS do not simply act as a polyanions and that sequence-specific protein-RNA interactions contribute to multivalency and promote phase separation. In combination with charge effects and the ability of a single RNA molecule to recruit multiple RBPs, RNA brings proteins into close proximity to promote phase separation (*19*). The importance of multivalent, sequence-specific RNA interactions to promote phase separation has also recently been noted for TDP-43 and hnRNPA1 (*24*–*26*). Our study shows that the interactions of RNA and RBPs can be similar in both dispersed and condensed phases. The data are in agreement with the widespread assumption that biomolecular structures remain largely unchanged within condensates (*3*). In addition, the data generally seem to support the conclusion that differences observed in iCLIP experiments for condensation mutants of TDP-43 are due to altered contacts mediated by the LCD and not due to general changes in the RNA binding interface (*27*). By using full-length proteins and mainly naturally occurring RNAs, we detect this consistency in the interface of protein and RNA for different types of RNA binding domains, including RRMs and IDRs. On the other hand, the data obtained for the nucleocapsid protein and PTBP1 suggest that unspecific RNA binding could also be favored under LLPS conditions. In contrast to published data (*48*), we could show that the nucleocapsid protein binds sequence-specifically to RNA in the dispersed state. However, we found that this sequence specificity is altered in condensates, where the interaction is potentially dominated by electrostatic contacts to the phosphate backbone. This observation is in line with previous observation of altered RNA binding within condensates (*29*).

Thus far, published structural differences inside condensates are rather subtle, including altered dynamics of RNA (*50*) and a slight compaction of the LCD of FUS (*16*). Our data illustrate an additional structural difference that can occur inside biomolecular condensates through intermolecular interactions that are less specific than in the dispersed state. Future studies will show whether the LLPS-CLIR-MS approach is also applicable to study phase separation of protein-RNA condensates with different material properties, such as hydrogels (*51*).

Limitations of the method include the coverage of the protein-RNA binding interface, which depends on the property to form a cross-link. Cross-link formation is influenced by three-dimensional (3D) conformation, as stacking of aromatic amino acids with bases is important for the formation of many cross-links, and nucleotide sequence, with uridine generally cross-linking most efficiently, although cytosine and guanine have also been found to cross-link frequently (*32*, *38*). Furthermore, the detection of cross-links depends on the protein sequence as peptides of suitable length need to be generated during protease digestion, and not all peptides ionize well. Similarly, RNA digestion conditions need to be comparable for the two phases, thereby resulting in similar lengths of RNA adducts, as otherwise mapping possibilities might be wrongly interpreted. Therefore, LLPS-CLIR-MS critically requires highly similar amounts of cross-linked protein-RNA complexes in samples of both phases. Last, the current CLIR-MS pipeline allows only a semiquantitative analysis of the data and serves mainly for the detection of qualitative differences. It is important to note that even under very favorable conditions, only identical cross-linking sites can be compared between samples in this way due to varying UV cross-linking efficiencies and ionization properties of peptides and peptide-RNA adducts.

The presented approach is expected to prove valuable for the structural characterization of protein-RNA complexes in the condensed phase, especially in combination with other liquid-state techniques, such as NMR and electron paramagnetic resonance (EPR) spectroscopy. NMR spectroscopy has been used to study IDRs in bulk phase or stabilized by agarose; however, thus far, folded domains could not be probed, but technical improvements to circumvent this limitation are likely to emerge (*16*). Long-range distances within biomolecular condensates have been successfully measured by EPR spectroscopy in agarose stabilized samples (*10*, *16*). As demonstrated before, protein-RNA cross-links can be used as intermolecular restraints for modeling of folded domains in complex with RNA (*32*, *34*, *52*). Therefore, LLPS-CLIR-MS, in combination with other techniques, should enable the structure determination of protein-RNA complexes within biomolecular condensates.

## MATERIALS AND METHODS

### Protein purification

Protein purification of PTBP1 (C250S/C251S) and RNA binding mutants was performed as described previously (*32*). Full-length FUS sequence was cloned into a pET24b vector by XhoI and BamHI restriction digestion and ligation that enables the translation of a tobacco etch virus (TEV)-cleavable GB1-His6-tagged protein. RNA binding mutants were generated by classical site-directed mutagenesis. FUS protein constructs were expressed in *Escherichia coli* BL21(DE3) overnight at 20°C after induction at an optical density of 0.6 with 0.1 mM isopropyl-β-d-thiogalactopyranoside (IPTG). Cells were harvested by centrifugation at 5000*g* and were directly resuspended in suspension buffer {50 mM 2-[4-(2-hydroxyethyl)piperazin-1-yl]ethanesulfonic acid (Hepes, pH 7.5), 150 mM NaCl, and protease inhibitors}. Twelve minutes of sonication on ice with 7 pulse-pause intervals at power level 70% were repeated twice to lyse the cells. The cell lysate was centrifuged at 15,000*g* at 4°C, and the resulting cell pellet was homogenized on ice (15 ml, Douncer homogenizer) in buffer A [8 M urea, 50 mM Hepes (pH 7.5), and 500 mM NaCl]. The homogenized protein solution was centrifuged again at 15,000*g* at 4°C for 25 min. The supernatant was collected and subjected to Ni-nitrilotriacetate (NTA) purification. The Ni-NTA (Qiagen) column was equilibrated with buffer A. After loading the filtered protein solution onto the column, the column was washed with 2 column volumes of buffer A and 4 column volumes of buffer A1 [1 M urea, 50 mM Hepes (pH 7.5), and 150 mM NaCl]. Protein was eluted by elution buffer [1 M urea, 50 mM Hepes (pH 7.5), 150 mM NaCl, and 250 mM imidazole]. TEV protease and 5 mM 2-mercaptoethanol (ME) were added into the eluted protein solution followed by a dialysis in buffer B [1 M urea, 50 mM Hepes (pH 7.5), 150 mM NaCl, 250 mM imidazole, and 5 mM ME] overnight, before a second dialysis in buffer C [6 M urea, 50 mM Hepes (pH 7.5), and 150 mM NaCl]. A second Ni-NTA purification was performed by applying the protein solution on a Ni-NTA column equilibrated in buffer C. Six column volumes of buffer C were used for washing. The flow-through of protein solution and washes were collected together and concentrated via centrifugation using 15 ml of Amicon UltraCentrifugal Filter Units (Millipore) with a molecular weight cutoff of 30 kDa. Full-length nucleocapsid construct was cloned into a pESPRIT vector between the Aat II and Not I cleavage sites with His_6_-tag and TEV protease cleavage sites at the N terminus (GenScript Biotech, The Netherlands). All nucleocapsid protein constructs were expressed in *E. coli* BL21 (DE3) overnight at 18°C after induction at an optical density of 0.6 with 0.6 mM IPTG. Cells were harvested by centrifuging at 3750*g* and resuspended in buffer containing 20 mM Tris-HCl (pH 8.0) and 1 M NaCl. The cells were lysed by cell cracking, and the lysate was centrifuged again at 17,000*g* at 4°C. The supernatant was subjected to standard Ni-NTA purification. Proteins were eluted with 20 mM Tris (pH 8.0), 500 mM NaCl, and 300 mM imidazole. Samples were then dialyzed against 20 mM Tris (pH 8), 300 mM NaCl, and 5 mM ME at 4°C overnight. Following TEV cleavage and removal of the excess N-terminal tag and TEV by Ni-NTA affinity, samples were concentrated and exchanged to 20 mM sodium phosphate (pH 6.0) and 50 mM NaCl (NMR buffer). For certain protein preparations when purity was not satisfactory, they were additionally subjected to size exclusion chromatography (Superdex 75/200) into the NMR buffer.

### RNA purification

3xUCUCU (5′-GGGAGAUCUCUAAAAAUCUCUAAAAAUCUCUAAAAA-3′), SL4 (5′-GGGGGACUGCGUUCGCGCUUUCCC-3′), SL34 (5′-GGGAUCCGAUUUCCCCAAAUGUGGGAAACUCGACUGCAUAAUUUGUGGUAGUGGGGGACUGCGUUCGCGCUUUCCCCUG-3′), and s2m (5′-GGUUCACCGAGGCCACGCGGAGUACGAUCGAGUGUACAGUGAACC-3′) were produced by in vitro run-off transcription with T7 RNA polymerase (purified in house) from two cDNA primers containing a T7 promoter or previously reported plasmids (*43*), respectively. Magnesium concentration was optimized for in vitro transcription reactions with both commercially available unlabeled nucleoside triphosphates (NTPs; Applichem) and ^13^C,^15^N–labeled NTPs (produced in house). The RNAs were purified by anion exchange chromatography in denaturing conditions (*53*). The purified RNA was precipitated by butanol extraction to eliminate urea and salts (*54*). Lyophilized RNA was resuspended in water or the respective buffers.

### Turbidity measurements

The turbidity (light scattering at 600 nm) of the samples was measured using a UV-visible spectrophotometer (ND-1000 Spectrophotometer, NanoDrop). For PTBP1, a defined volume of protein was diluted in PTBP1 buffer [10 mM sodium phosphate (pH 6.5) and 20 mM NaCl] to obtain a specific protein concentration in a total reaction volume of 10 μL. The RNA (1 μl) was additionally mixed with PTBP1 buffer to obtain a 10× concentration of the final RNA concentration required in the phase separation sample. The 10× RNA sample (1 μl) was added to the protein in PTBP1 buffer (9 μl), and the sample was mixed five times with a pipette and incubated for 1 min at room temperature before absorbance measurement at 600 nm. For measurements of FUS, 20 μM protein was prepared in turbidity buffer [5 mM potassium phosphate (pH 6.0), 100 mM KCl, 2 μM ZnCl_2_, and 1 mM tris(2-carboxyethyl)phosphine (TCEP)]. Similarly, RNAs were prepared at defined concentrations in the turbidity buffer before the addition of the protein solution. An analogous setup was used for nucleocapsid comprising 20 μl of reaction volumes, fixed protein concentration of 60 μM, and varying amount of s2m RNA. Here, NMR buffer was used for turbidity measurements. All turbidity measurements presented in the paper were the average values of multiple (≥3) measurements. Average values are presented together with SD of the measurements. All turbidity data values are shown in table S2.

### Microscopy

To investigate the phase separation behavior of different RNAs with various PTBP1 constructs, the samples were investigated by bright-field microscopy (Olympus CKX41). Thereby, a defined volume of protein was diluted in PTBP1 buffer [10 mM sodium phosphate (pH 6.5) and 20 mM NaCl] to obtain a specific protein concentration in a total reaction volume of 10 μl. The sample was prepared on a well plate. The RNA (1 μl) was additionally mixed with PTBP1 buffer to obtain a 10× concentration of the final RNA concentration required in the phase separation sample. The 10× RNA sample (1 μl) was added to the protein in PTBP1 buffer (9 μl), and the sample was mixed five times with a pipette. After a time frame of 3 min, a microscopy image using a 20× microscope objective lens (Olympus LCAch N 20×/ 0.40 PhC ∞/1 FN 22) was taken. Similar setups were used for FUS (20 μl of sample and 20 μM protein with RNA in turbidity buffer) and nucleocapsid.

### NMR spectroscopy

Titration of nucleocapsid with s2m-polyA involving 1D ^1^H detection of nucleocapsid, 1D ^13^C-edited, and ^13^C-^1^H HSQC experiments of ^15^N/^13^C labeled s2m-polyA spectra were acquired at 303 K on a 900-MHz Avance 3 HD Bruker spectrometer equipped with a TCI cryo-probe. ^1^H-^1^H total correlation spectroscopy spectra of s2m-polyC RNA were acquired at 310 K on a 500-MHz Avance NEO Bruker spectrometer equipped with a QCI cryo-probe. DOSY experiments were performed as described previously (*16*).

### Cross-linking of isotope labeled RNA coupled to mass spectrometry

For PTBP1, per replicate, a reaction volume of 1-ml sample containing PTBP1 full-length (30 μM) and an equimolar amount of unlabeled 3xUCUCU (5 μM) and ^13^C-^15^N-labeled 3xUCUCU (5 μM) in PTBP1 buffer [10 mM sodium phosphate (pH 6.5), 20 mM NaCl, and 1 mM TCEP] was prepared in a 1.5-ml microcentrifuge tube. To UV cross-link PTBP1 and 3xUCUCU, each 1-ml sample was divided into 10 wells with 100 μl per well volume on a 96-well plate. All samples were irradiated at an energy of 2.4 J/cm^2^ (Spectrolinker XL-1500 UV Cross-linker, Spectronics Corporation). The 10 aliquots (100 μl) were pooled in a 1.5-ml Eppendorf tube and subsequently centrifuged (5 min, 5000*g*, room temperature). Under these centrifugation conditions, the dispersed phase corresponded to an estimated volume of 990 μl and the condensed phase to a volume of 10 μl. The diluted phase was removed, and the condensed phase was resuspended in PTBP1 buffer containing urea [10 mM sodium phosphate (pH 6.5), 20 mM NaCl, and 3 M urea] to a volume of 100 μl (10× dilution of condensed phase). By measuring the absorbance at 260 nm using a UV-Vis spectrophotometer (ND-1000 Spectrophotometer, NanoDrop) and calculating the molar extinction coefficients, the concentration of the 10× dilution of the condensed phase as well the diluted phase was determined using Beer-Lambert law. Considering the calculated concentrations and the respective volume in each phase, the amount of RNA in the condensed and diluted phase was determined. The RNA concentration in the diluted phase was calculated by dividing the calculated RNA concentration of the diluted phase by the original RNA concentration in the sample. Equal sample amounts were confirmed by SDS–polyacrylamide gel electrophoresis (SDS-PAGE) analysis.

For FUS, induction of phase separation was achieved at an RNA/protein ratio 0.075:1 (Fig. 3) and 0.1:1 (fig. S5) at 20 μM FUS concentration in 3 × 500 μl FUS droplet buffer [5 mM potassium phosphate (pH 6.0), 100 mM KCl, 2 μM ZnCl_2_, and 1 mM TCEP]. UV irradiation was performed at an energy of 0.8 J/cm^2^. Samples were centrifuged at 5000*g* for 5 min, followed by the removal of the dispersed phase, the condensed phase sample was resuspended in 6 M urea containing FUS protein storage buffer [50 mM HEPES (pH 7.5), 150 mM NaCl, and 6 M urea]. Similarly, for nucleocapsid, samples were prepared at an RNA/protein ratio 0.2:1 in NMR buffer at 100 μM N protein concentration and irradiated at 4.8 J/cm^2^. The samples were centrifuged, and the condensed phase was resuspended in NMR buffer supplemented with 4 M urea.

After ethanol precipitation, the pellets of all samples were resuspended in 50 μl Tris-HCl with urea [50 mM (pH 7.9), 4 M urea] and further diluted with 150 μl Tris-HCl [50 mM (pH 7.9)]. Sample amounts were determined by absorbance measurement and SDS-PAGE analysis. The mass of cross-linked sample was calculated on the basis of the assumption that the molar ratio of RNA to protein in the protein-RNA complex was 1:1. The RNA was digested using ribonuclease T1 (RNase T1) (Thermo Fisher Scientific) and RNase A (Ambion) at 52°C for 2 hours (0.5 U of RNase T1 and 0.5 μg of RNase A per 1 nmol of RNA-protein sample). For PTBP1 and FUS, RNA was further digested using benzonase (Sigma-Aldrich) for 1.5 hours at 37°C (62.5 U per 1 nmol sample). To digest the protein, the samples were incubated with trypsin at 24:1 to 40:1 ratios (Promega) at 37°C overnight, and the reaction was inactivated the following morning at 70°C for 10 min. Nucleocapsid samples were additionally processed for postdigestion labeling as described previously, omitting the benzonase digestion (*47*).

Samples were purified using solid phase extraction (SepPak 100 mg tC18 cartridges, Waters) and dried by vacuum centrifugation. Enrichment of protein-RNA cross-links by metal oxide affinity chromatography was performed as described previously (*32*–*34*). The enriched samples were cleaned up using STAGE tip C_18_ solid phase extraction (*55*) following a previously described procedure (*33*, *34*, *38*). Afterward, samples were dried by vacuum centrifugation and resuspended in 20 μL of 5% acetonitrile with 0.1% formic acid. Five microliters of each sample was injected for LC-MS/MS analysis, and each sample was analyzed in duplicate (except otherwise noted). LC-MS/MS analyses were conducted using an Easy-nLC 1200 HPLC system (Thermo Fisher Scientific), connected to an Orbitrap Fusion Lumos mass spectrometer (Thermo Fisher Scientific) with a Nanoflex nanoflow electrospray ionization source (Thermo Fisher Scientific). Conditions for separation of peptide-RNA adducts by liquid chromatography and mass spectrometer settings were reported previously (*33*, *34*, *38*).

MS data analysis was performed with xQuest (version 2.1.5) (*56*) and RNxQuest (*57*), both available from https://gitlab.ethz.ch/leitner_lab/. Data processing was performed essentially as described previously for ^13^C-^15^N labeled, in vitro transcribed RNAs, or postdigestion labeled samples (*33*, *34*, *38*, *47*, *57*). All results (provided in table S1) were filtered to 1% false discovery rate at the level of unique combinations of peptide sequence, RNA adduct mass, and modification site. Identifications from xQuest were plotted using functions from the RNxQuest Python package and custom Python 3.7 scripts.
