# Supplementary material for: Specific protein-RNA interactions are mostly preserved in biomolecular condensates
Source: Sci Adv. 2024 Mar 6;10(10):eadm7435. doi: 10.1126/sciadv.adm7435 (PMC10917357; doi:10.1126/sciadv.adm7435)
Supplement: Supplementary file 1 — Figs. S1 to S7 Legends for tables S1 and S2 [file sciadv.adm7435_sm.pdf]

Science Advances
AAAS

Supplementary Materials for

Specific protein-RNA interactions are mostly preserved in biomolecular condensates

Tebbe de Vries *et al.*

Corresponding author: Frédéric H.-T. Allain, allain@bc.biol.ethz.ch; Alexander Leitner, leitner@imsb.biol.ethz.ch

Sci. Adv. **10**, eadm7435 (2024)
DOI: 10.1126/sciadv.adm7435

The PDF file includes:

Figs. S1 to S7
Legends for tables S1 and S2

Other Supplementary Material for this manuscript includes the following:

Tables S1 and S2

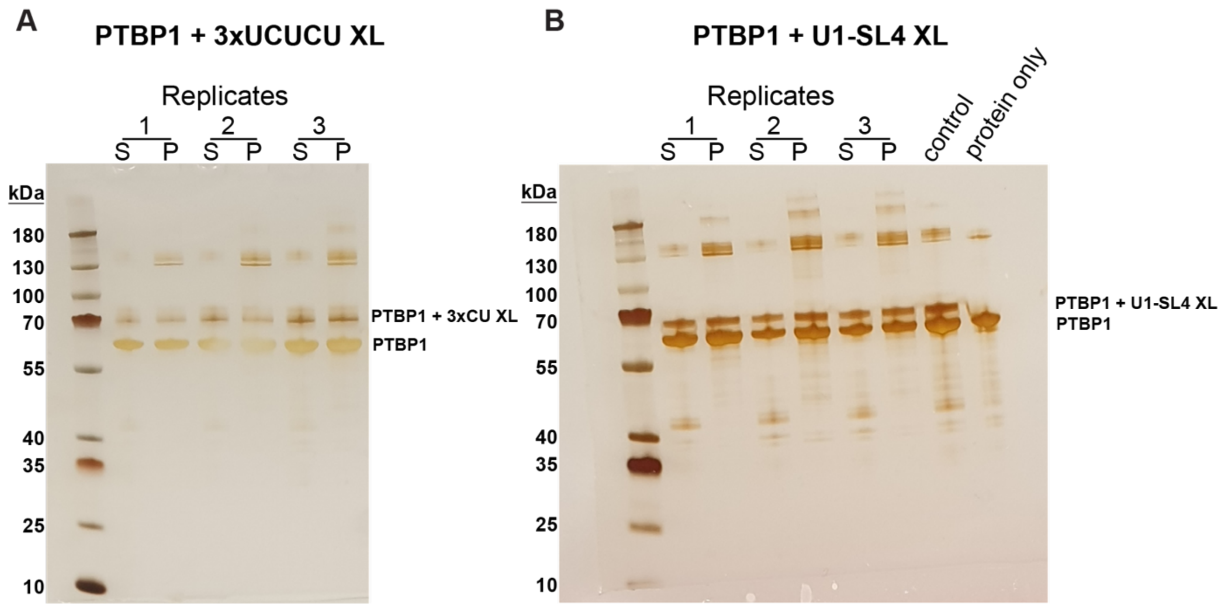

Fig. S1. The presence of cross-linked protein-RNA complexes in both phases confirmed by SDS gel analysis. Gels show three replicates of (**A**) 30 μM PTBP1 with 10 μM 3xUCUCU and (**B**) 30 μM PTBP1 with 10 μM U1-SL4 RNA. S stands for supernatant corresponding to the dispersed phase and P for pellet of the condensed phase formed after centrifugation.

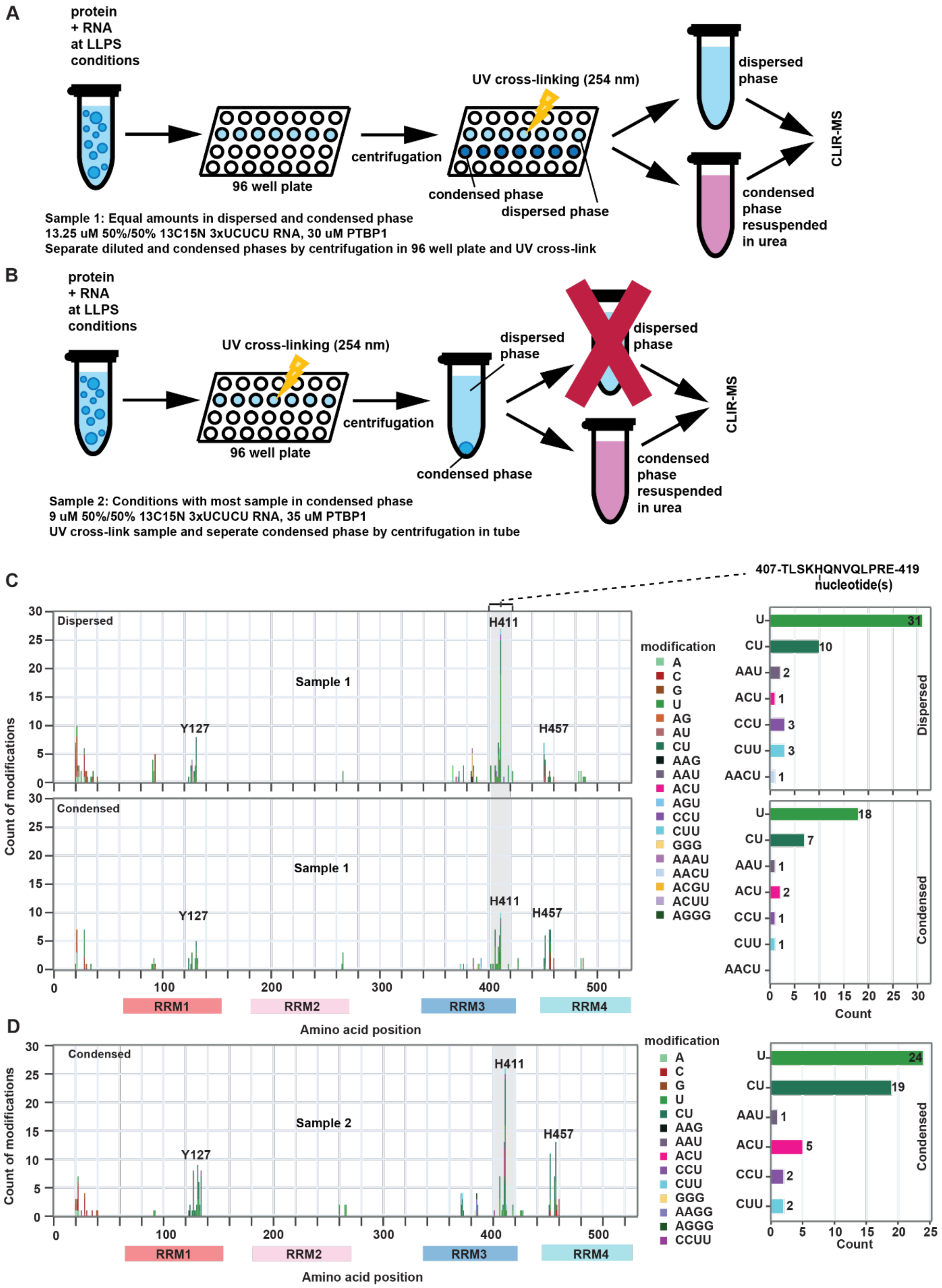

Fig. S2. Both simultaneous and individual UV cross-linking suggest similar RNA binding behavior between dispersed and condensed phase. (**A**) Alternative UV cross-linking setup, in which both phases are separated by centrifugation prior to UV cross-linking and subsequent CLIR-MS analysis (Sample 1). (**B**) The standard UV cross-linking setup with a sample that contains mainly material in the condensed phase (Sample 2). The mass spectrometric

analysis of the cross-linked products is "peptide-centric" in the sense that short oligonucleotides are identified as modifications to a certain peptide sequence. The isotope labelling of the RNA introduces a unique doublet pattern (light/heavy isotopes) only for peptides carrying an RNA cross-link, not for unmodified peptides. This way, only MS/MS spectra originating from doublet signals need to be searched, which speeds up the data analysis and reduces the error rate. If the protein would be labelled, this would not lead to such benefits for data analysis. (**C**) PTBP1 and 3xUCUCU sample prepared using the setup depicted in (**A**). (**D**) PTBP1 and 3xUCUCU sample prepared using the setup depicted in (**B**).

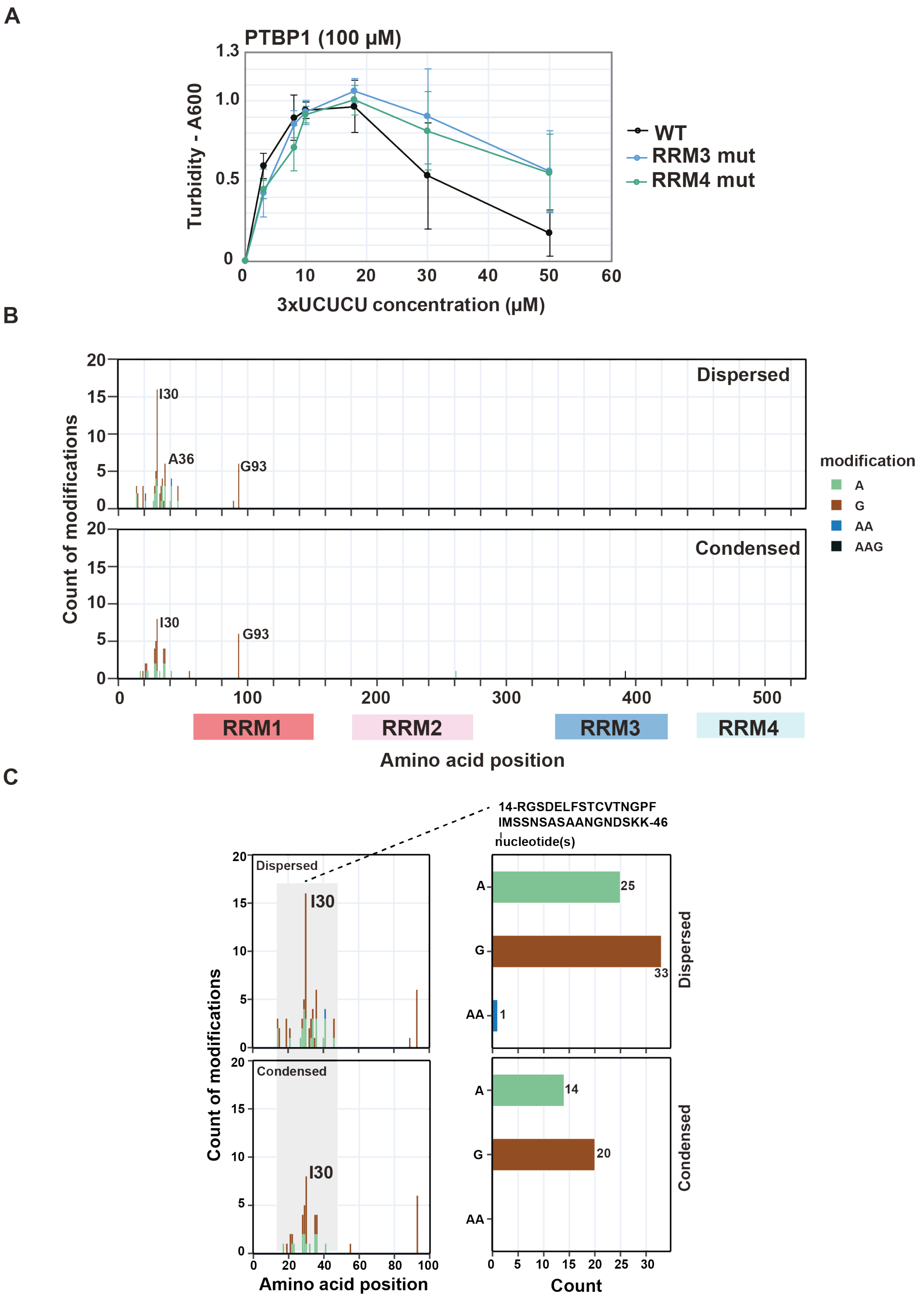

Fig. S3. PTBP1 exhibits only unspecific cross-links to 3xAGAGA RNA in both condensed and dispersed phase. (**A**) Turbidity measurements of PTBP1 RRM3 and RRM4 mutants and 3xUCUCU compared to wild-type (WT). (**B**) LLPS-CLIR-MS results of PTBP1 and 3xAGAGA at an RNA/protein ratio 0.1:1. (**C**) Amino acid sequence near NLS as a main cross-linking site of PTBP1 and 3xAGAGA with highlighted nucleotide modifications detected in the experiment.

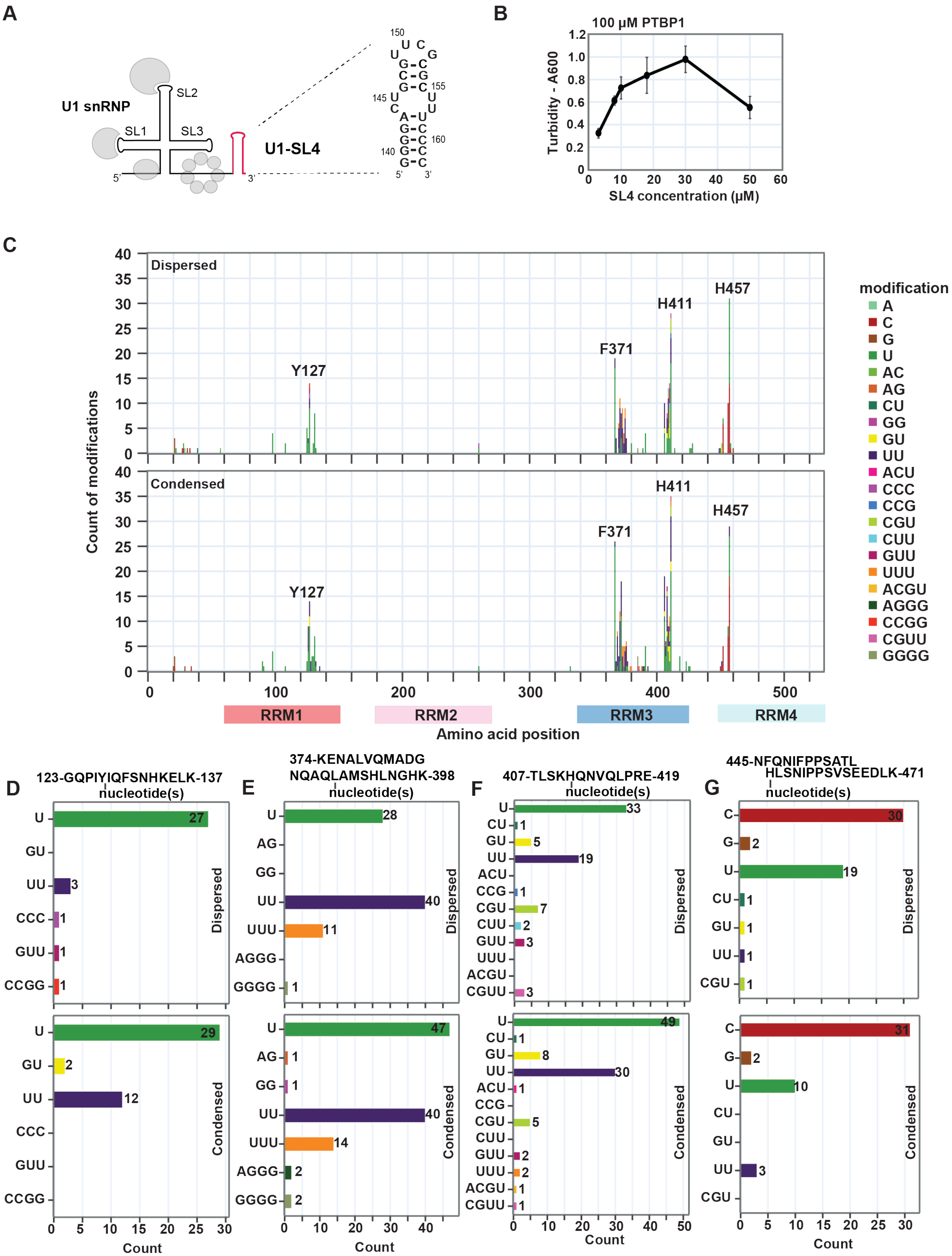

Fig. S4. PTBP1 binding to U1-SL4 is unaltered in the condensed state compared to the dispersed state. (**A**) Scheme of SL4 of U1 snRNA (U1-SL4). (**B**) Turbidity measurement (absorbance at 600 nm) of PTBP1 and U1-SL4. (**C**) LLPS-CLIR-MS results of PTBP1 U1-SL4 with concentrations of 30 µM and 10 µM, respectively. (**D-G**) Mapping of PTBP1 on U1-SL4 sequence based on the detected nucleotide sequences.

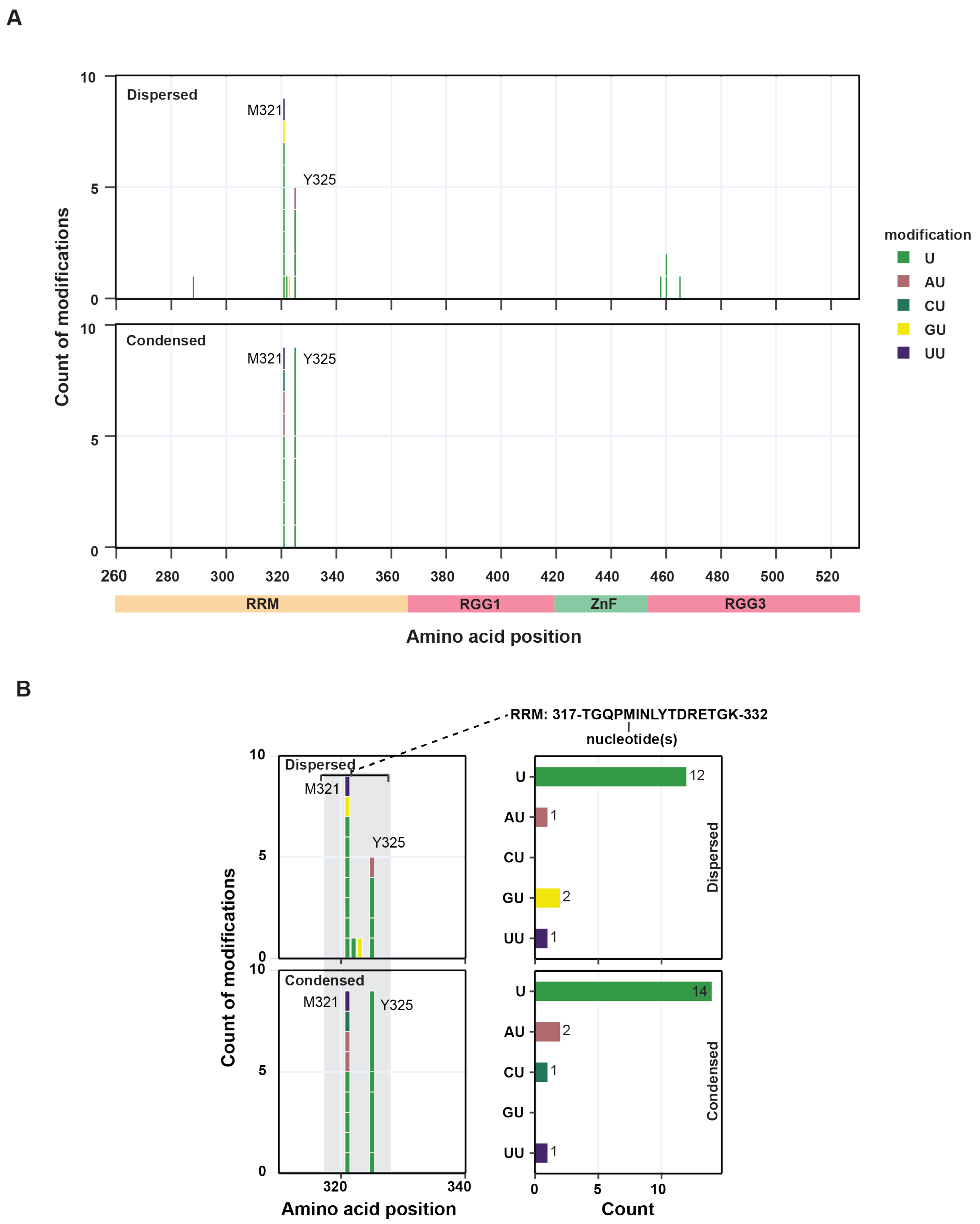

Fig. S5. FUS RRM sequence-specific cross-links to U1-SL34 are preserved in both condensed and dispersed phase. (**A**) LLPS-CLIR-MS results of FUS U1-SL34 at an RNA/protein ratio 0.1:1 at 20 µM FUS concentration. (**B**) Main cross-linking site of FUS RRM (M321 and Y325) and highlighted nucleotide modifications detected in the experiment.

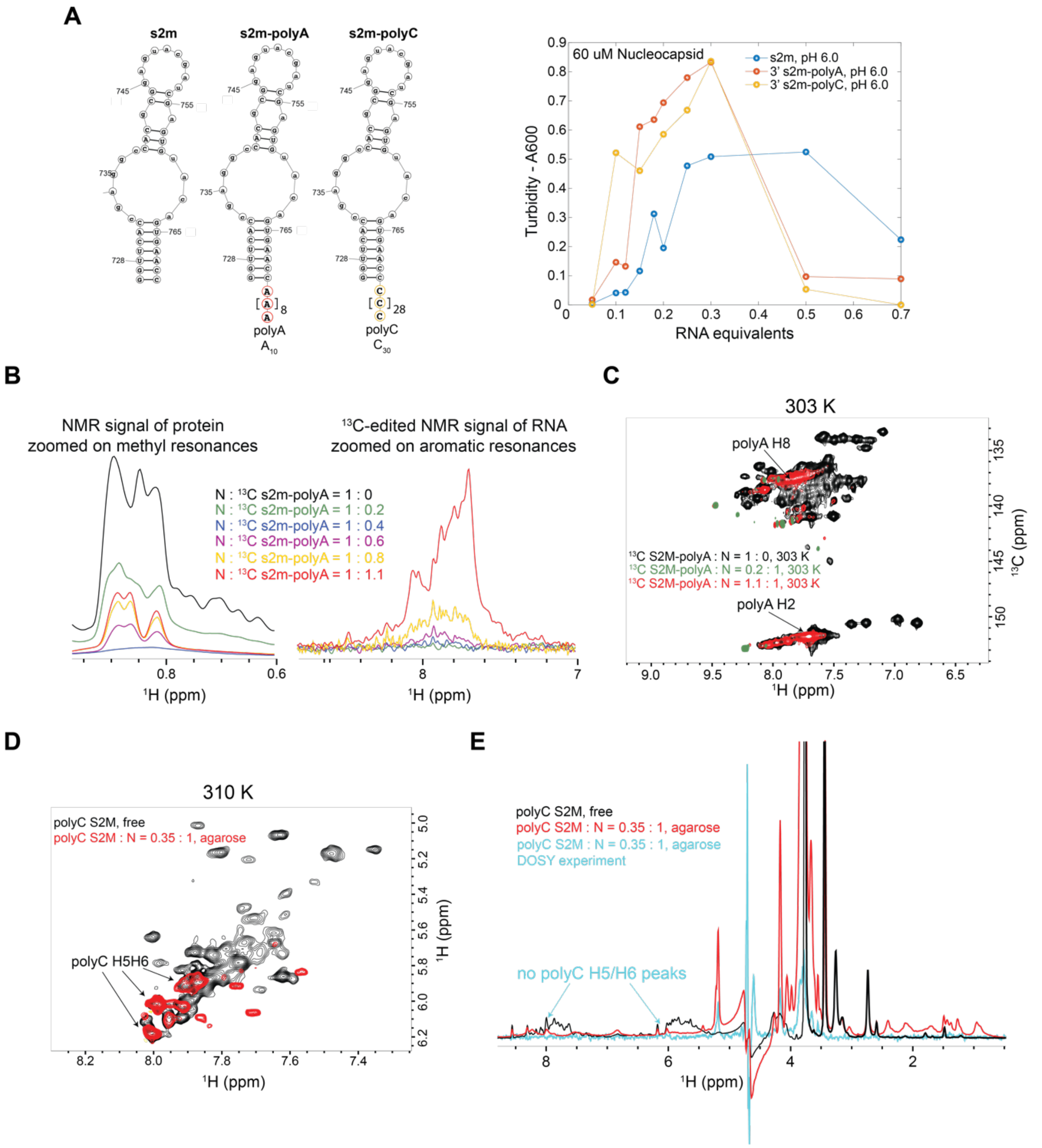

Fig. S6. Unspecific interactions of Nucleocapsid are promoted in the condensed phase. (**A**) To explore the specificity of Nucleocapsid-RNA interactions, s2m RNA was extended on its 3' end by 10 nucleotides long polyA or a 30 nucleotides long polyC tail (referred to as s2m-polyA and s2m-polyC, respectively). Turbidity measurements show that the addition of the polyA/polyC tail to the 3' end of s2m substantially influences the phase separation of full-length Nucleocapsid. Larger turbidity upon addition of smaller equivalents of RNA implies an involvement of the tails in LLPS. (**B**) NMR observation of Nucleocapsid and s2m-polyA during the protein titration with RNA. The protein signal almost completely broadens between 0.2 and 0.4 equivalents of RNA that corresponds to the maximum turbidity, suggesting the strongest interactions and slowest tumbling of the complex at these stoichiometries. As RNA concentration is increased and consequently the droplets dissolve, the protein signal becomes more dynamic and therefore observable by NMR. Correspondingly, all the RNA peaks were unobservable while the RNA was in substoichiometric ratios that promoted LLPS. Note the disproportional multi-fold increase in signal intensity from 0.8 to 1.1 eq. of added RNA when the droplets were dissolved. (**C**) ^{13}C-^{1}H HSQC aromatic spectra of RNA show that adenine H2 and H8 peaks of the polyA tail are first to be observed upon dissolution of the droplets (1.1 equivalent of RNA, red). In the LLPS regime (0.2 equivalents of RNA, green), the polyA tail is not observable. This suggests that

the polyA tail was tightly packed and tumbling very slowly while Nucleocapsid was in LLPS regime, hence not observable by NMR spectroscopy. After dissolving the droplets, the polyA tail starts to tumble fast in the solution and becomes observable. (**D**) ^{1}H-^{1}H TOCSY spectrum showing the H5-H6 proton correlations of free s2m-polyC (30C-long tail) and Nucleocapsid bound s2m-polyC in 0.5% agarose (agarose is added to stabilize droplets not allowing them to sediment (10)). After 15 hours of signal averaging, polyC H5/H6 proton correlations were detected, while no correlations corresponding to the s2m sequence were observed. (**E**) A DOSY experiment and application of a strong gradient (10) resulted in the absence of H5/H6 proton peaks. This implied that polyC H5/H6 proton correlations detected by ^{1}H-^{1}H TOCSY stem from the dispersed phase, thus confirming that the polyC tail is observable in dispersed phase when specific protein-RNA interactions dominate. In the droplet phase H5/H6 proton signals of the polyC tail are most likely unobservable because unspecific interactions are promoted.

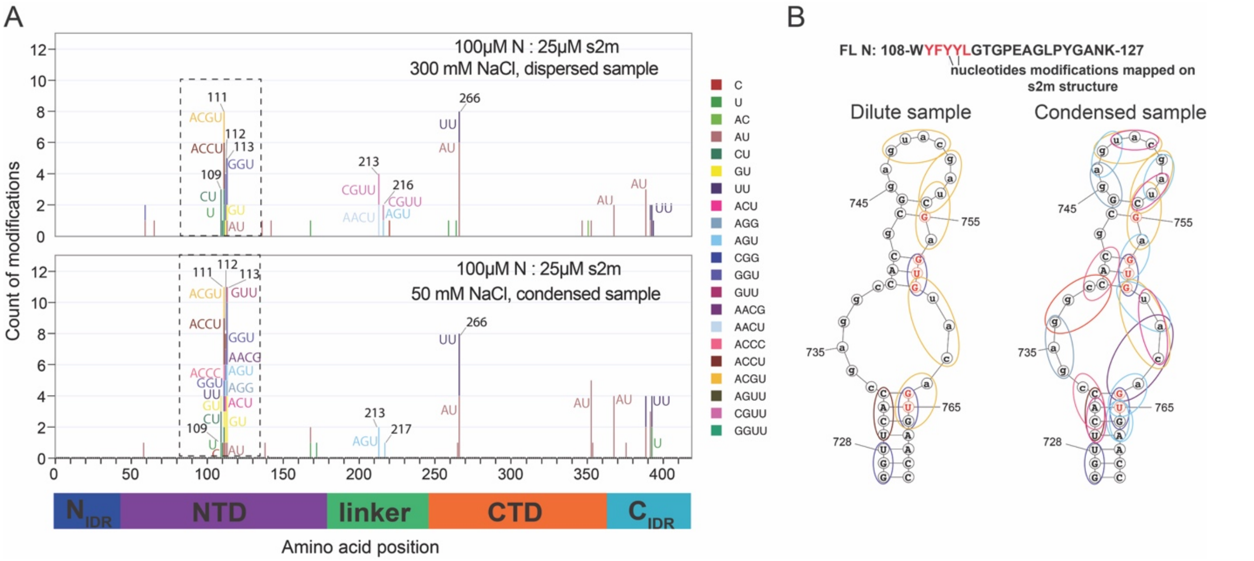

Fig. S7. CLIR-MS experiments with samples mimicking the dispersed and condensed phases confirm less specific interactions in Nucleocapsid protein condensates. (**A**) While high salt concentration (300 mM NaCl) dissolves the Nucleocapsid condensates, at 50 mM NaCl concentration and 0.25 equivalents of s2m, most of the protein and RNA are in the condensed phase. Overview of the protein-RNA cross-links detected for Nucleocapsid protein and s2m in these two samples mimicking purely dispersed and condensed phases. (**B**) Mapping of the RNA-binding site of Nucleocapsid NTD on s2m RNA. Ellipsoids on the s2m secondary structure represent detected cross-linked nucleotide modifications and are color-coded according to (**A**). The combination of cross-linked nucleotides corroborates an altered interaction of the NTD with s2m in the condensed state.

Supplementary Table 1. Summary of MS identifications obtained by the LLPS-CLIR-MS method.

Supplementary Table 2. Raw data representing technical replicates in turbidity measurements.
